# Exploring the association between regional fat distribution and atrial fibrillation risks: a comprehensive cohort study

**DOI:** 10.3389/fendo.2024.1367653

**Published:** 2024-03-22

**Authors:** Chenkai Wu, Yuefei Xu, Zhenhua Xing

**Affiliations:** ^1^ Department of Cardiology, The Second Xiangya Hospital, Central South University, Changsha, Hunan, China; ^2^ Department of Emergency Medicine, Second Xiangya Hospital, Central South University, Changsha, Hunan, China; ^3^ Emergency Medicine and Difficult Diseases Institute, Central South University, Changsha, Hunan, China

**Keywords:** fat mass distribution, total fat mass, atrial fibrillation, visceral adipose tissue, abdominal subcutaneous adipose tissue

## Abstract

**Background:**

The contribution of total fat mass and regional fat distribution to the risk of AF has rarely been studied.

**Methods:**

This prospective cohort study(N=494,063) evaluated the association of total fat mass measured by fat percentage (FP) and regional fat measured by arm fat percentage (AFP), trunk fat percentage (TFP), and leg fat percentage (LFP) with incident AF. A subgroup (N = 25,581) underwent MRI, which allowed us to further assess whether visceral adipose tissue (VAT) and abdominal subcutaneous adipose tissue (ASAT) of the trunk fat exert different effects on AF incidence.

**Results:**

Over, a median 12.9 ± 1.86 years of follow-up, 29,658 participants (cumulative rate: 6.0%) developed AF. Each 1-standard deviation (SD) increase in LFP was associated with a 16% lower risk of AF (HR: 0.84, 95% CI: 0.82, 0.85). The association between FP and AF was weaker than that between LFP and AF (HR: 0.90, 95% CI: 0.89, 0.92). AFP and TFP only had a marginal association with a lower incidence of AF. Both the VAT and ASAT showed a U-shaped relationship with incident AF.

**Conclusions:**

Fat mass, mainly leg fat mass, was associated with a lower risk of AF. ASAT did not exert protective effects.

## Introduction

The incidence of atrial fibrillation (AF) has surged globally and poses significant risks, including dementia, stroke, and heart failure (HF), impacting health-related quality of life (1–3). Therefore, identifying modifiable risk factors for AF is extremely important for its prevention. Among these, obesity is recognized as a major risk factor. Common obesity-related anthropometric measures, such as body mass index (BMI), waist circumference (WC), and waist-to-hip ratio, are associated with AF, likely due to inflammation and metabolic disorders induced by obesity (4). Obesity can further lead to increased LV filling pressures and LA pressure due to the increased volume of adipose tissue, which is a well-established risk factor for AF (4).

However, recent studies have challenged this hypothesis (5, 6). Lean body mass (LBM) emerges as a primary anthropometric risk factor for AF, challenging the clarity of the association between obesity and AF (6). Frost et al. observed that both LBM and fat mass correlate with a higher AF risk, with fat mass showing a correlation to LBM (4). Furthermore, recent studies have demonstrated that obesity is a heterogeneous and complex condition and that regional fat mass may contribute differently to the development of obesity-related diseases such as AF (7). For instance, trunk fat mass links significantly to metabolic complications and inflammation like cardiovascular diseases (CVD) and type 2 diabetes, whereas leg fat mass (LFM) exhibits a protective effect (8, 9).

Despite these insights, previous studies on regional fat mass effects on AF primarily focused on inflammation and complications induced by excess trunk mass (measured by WC), overlooking the potential protective role of LFM (4, 10). While dual-energy X-ray absorptiometry (DEXA), magnetic resonance imaging (MRI), and computed tomography (CT) are preferred methods for accurate fat mass and LBM measurement, their practicality is limited in large cohort studies and routine clinical practice due to accessibility, radiation, and cost concerns. Bioimpedance analysis (BIA) emerges as a simple and effective alternative for measuring fat mass, offering accuracy comparable to DEXA (11). In this context, we aimed to evaluate the association between total and regional fat mass (arm fat, trunk fat, and leg fat) measured by BIA and the risk of incident AF, aiming to provide a comprehensive understanding of these relationships.

## Methods

### Study design and population

The Biobank study, a population-based prospective cohort study conducted in the UK between 2006 and 2010, encompassed over 500,000 residents aged 40–69 years. The study’s design has been previously outlined (12). BIA measurements did not distinguish between visceral adipose tissue (VAT) and abdominal subcutaneous adipose tissue (ASAT). However, VAT and ASAT may have different roles in metabolism and inflammation (7). A subgroup of the Biobank study underwent MRI scanning (N = 25,581), which provided us the opportunity to assess the different roles of VAT and ASAT in AF. Ethical approval for the Biobank study was granted by the Northwest Multi-Center Research Ethics Committee(11/NW/0382), and all participants provided written informed consent. Comprehensive details about this study are available at www.ukbiobank.ac.uk. Participants with AF at baseline were excluded from this study.

### Exposure and covariates

Body weight was measured and segmental single-frequency BIA was performed using a Tanita BC-418 MA (Tanita Corporation, Arlington Heights, IL, USA). Through BIA, measurements of total and regional fat mass (leg, arm, and trunk) were obtained. Height, measured using the Seca 202 scale (Seca, Hamburg, Germany) without shoes, was utilized to calculate Body Mass Index (BMI), an indicator of general obesity (weight in kilograms divided by the square of height in meters).Regional fat percentage, specifically arm fat percentage (AFP), trunk fat percentage (TFP), and leg fat percentage (LFP), served as indicators of regional fat. These percentages were calculated as fat mass divided by total mass for the corresponding body parts (arm, trunk, and leg). Fat percentage (FP) was used as an indicator of total fat mass, calculated as total fat mass divided by total weight (11). The volumes of the VAT, ASAT, and total trunk fat (TTF) were calculated based on previous studies (13).

Baseline data on participants’ demographic and clinical characteristics, encompassing lifestyle and health information, medical history, and biological samples, were acquired through physical examinations, interviews, and laboratory tests. These included age, sex, race, BMI, history of myocardial infarction, HF, and diabetes, smoking and alcohol consumption, diet, physical activity, systolic blood pressure (SBP), diastolic blood pressure (DBP), and medication for hypertension. History of myocardial infarction, diabetes, HF, and hypertension medication use was identified using questionnaires, interviews, and medical records. Physical activity was measured using the metabolic equivalent task (MET) based on the International Physical Activity Questionnaire (IPAQ). Diet score (ranging from 0 to 4) was calculated based on the following diet factors: cooked or raw vegetable intake ≥ four tablespoons/day; fresh fruit intake ≥ three pieces/day; fish intake ≥ twice/week, and processed meat intake ≤ twice/week. SBP and DBP were measured at the assessment center by registered nurses.

### Definition of AF

In the current analysis, atrial flutter was considered equivalent to atrial fibrillation (AF). The diagnosis of AF (ICD10 code I48) was established through diagnostic codes linked to hospital encounters and death records. Baseline and incident AF cases were identified based on the “first occurrence of health outcomes defined by a three-character International Statistical Classification of Diseases and Related Health Problems 10th Revision code” (field ID 131351). The follow-up period for each participant was determined from the date of attending the assessment center (between 2006 and 2010) to the date of AF diagnosis, death, or censoring (May 31, 2022, field ID 131350), whichever occurred first.

### Statistical analysis

The baseline characteristics of the included participants are presented as mean ± standard deviation (SD) with normal distribution or as median (25th and 75th percentiles) with skewed distribution. The continuous variables were compared using Student’s t-test or Mann–Whitney U test according to the distribution type, and the categorical variables were compared using chi-square tests. Missing data were coded as missing indicator categories for categorical variables, such as race, and mean values for continuous variables. The correlations between total and regional fat mass percentage or total and regional fat percentage were assessed using Pearson’s coefficient. The relationship between total and regional fat mass percentage/index, treated as both continuous and categorical variables (Q1, Q2, and Q3), and the risk of atrial fibrillation (AF) was evaluated using the Cox proportional-hazard model. Model 1 was adjusted for age, race, sex, and BMI. Model 2 was adjusted for age, race, sex, myocardial infarction (yes/no), diabetes (yes/no), HF (yes/no), hypertension medication use (yes/no), diet score (0–4), smoking and alcohol consumption, MET, SBP, DBP, and BMI. Both models were adjusted for BMI, which accounts for the fact that participants with the same fat mass who differ in BMI will have totally different fat distributions (4). Restricted cubic splines with four knots at the 5th, 35th, 65th, and 95th percentiles were used to explore the dose-response association between the total and regional fat mass percentage and incident AF. We found no evidence of violation of the proportional hazard assumption based on tests using Schoenfeld residuals. Discrimination in the models was assessed using Harrell’s C-statistic.

Sensitivity analysis was performed to verify the robustness of our results. First, arm, trunk, and leg fat were normalized for height by dividing by the square of height in meters, similar to the calculation of BMI. This normalization was implemented to address the variability in body composition among participants with the same weight and regional fat mass but different heights (11).we used total and regional fat mass indicators (arm fat index [AFI], trunk fat index [TFI], and leg fat index [LFI]) instead of fat mass percentage to reevaluate the association between total and regional fat mass and incident AF. Additionally, to avoid bias in the Cox proportional-hazard model, we used competing risk models (modeling sub-distributional hazard ratios) for HF. Competing events of all-cause mortality, as a permanent condition, may prevent the occurrence of AF. Furthermore, we utilized propensity score matching to assess the connection between total and regional fat percentage and AF. This involved employing a 1:1 nearest-neighbor matching approach without replacement and setting a matching tolerance (caliper) of 0.05. The goal was to ensure balanced baseline characteristics between the groups. To generate the propensity score, we employed a logistic regression model, with AF as the outcome variable and all potential confounding factors listed in Model 2 and compared total and regional fat percentage using t test. Subgroup analyses were conducted according to age (<60 and ≥60 years), sex, race, smoking status, and history of myocardial infarction, and obesity categories (non-obese <30 kg/m² and obese ≥30 kg/m²) and HF.

All statistical analyses were two-sided, and a *P*-value <0.05 was considered statistically significant. All analyses were performed using the STATA 17 software (StataCorp) between April 1, 2022 and June 31, 2022.

## Results

### Baseline characteristics of included participants

Of the 502,461 participants in the Biobank study, 8,398 were excluded from the present study for the following reasons: baseline AF, lack of total or regional fat data, and loss of follow-up. A total of 494,063 participants (54.8% female, 88% white, 5.1% with a history of diabetes, and 2.1% with myocardial infarction) remained for further analysis. The included participants had an average age of 56.4 ± 8.1 years. Over a median follow-up period of 12.9 ± 1.86 years, 29,658 participants (4.64 per 1000 persons per year) developed AF. **Table 1** presented the baseline characteristics of the participants based on the incidence of AF. Participants who developed incident AF tended to have higher BMI, SBP, and DBP and lower diet scores. They were also more likely to be older, male, non-white, current smokers, had a history of diabetes, HF, and myocardial infarction, and consumed more cigarettes and alcohol. **Supplementary Figure 1** displays the correlation of FP, AFP, TFP, LFP, AFI, TFI, and LFI (all P < 0.01).

**Table 1:** Characteristics of included participants.

N	Incidence AF		P-value
	No(N=464,405)	Yes(N=29,658)	
**FP (%)**	31.5 ± 8.53	31.6 ± 8.54	<0.01
**AFP (%)**	30.1 ± 10.2	30.0 ± 29.8	0.15
**TFP (%)**	31.1± 8.00	32.8 ± 8.01	<0.01
**LFP (%)**	32.7 ± 10.6	30.7 ± 10.9	<0.01
**BMI (Kg/m^2^)**	27.31 ± 4.73	28.99 ± 5.42	<0.01
**Age (years)**	56.09 ± 8.08	61.90 ± 6.08	<0.01
**Sex, male (%)**	205,044 (44.15%)	18,218 (61.43%)	<0.01
**Race, White (%)**	408,349 (87.93%)	27,049 (91.20%)	<0.01
**Smoker (%)**			<0.01
**Never**	256,472 (55.23%)	13,232 (44.62%)	
**Current**	156,239 (33.64%)	12,889 (43.46%)	
**Quit**	49,036 (10.56%)	3,310 (11.16%)	
**Alcohol, times (%)**			<0.01
**Daily**	92,594 (20.00%)	7,122 (24.10%)	
**3-4/week**	107,141 (23.14%)	6,490 (21.96%)	
**1-2/week**	120,345 (25.99%)	7,029 (23.78%)	
**1-3/month**	52,258 (11.29%)	2,798 (9.47%)	
**Occasionally**	53,645 (11.59%)	3,457 (11.70%)	
**never**	37,056 (8.00%)	2,657 (8.99%)	
**Diet Score**			0.04
0	6,143 (1.36%)	304 (1.14%)	
1	54,391 (12.09%)	3,278 (12.28%)	
2	138,650 (30.81%)	8,400 (31.46%)	
3	163,569 (36.34%)	9,585 (35.89%)	
4	87,311 (19.40%)	5,137 (19.24%)	
**Diabetes (%)**	22,276 (4.80%)	3,121 (10.52%)	<0.01
**Heart failure (%)**	1,344 (0.29%)	462 (1.56%)	<0.01
**Myocardial infarction (%)**	8,533 (1.84%)	2,002 (6.75%)	<0.01
**Previous stroke (%)**	3250 (0.70%)	201 (0.68%)	0.39
**Previous cancer (%)**	3954 (0.85%)	258 (0.86%)	0.10
**MET (min/week)**	965 ± 941	974 ± 952	0.57
**C2HEST score (IQR)**	1 (0-1)	1 (0-1)	0.75
**Stroke (%)**	5,451 (1.1%)	315 (1.1%)	0.09
**Medication for hypertension (%)**	415,710 (89.51%)	21,557 (72.69%)	<0.01
**MET (min/week)**	1,041.15 ± 974.25	1,030.27 ± 949.13	0.06
**DBP (mmHg)**	82.17 ± 10.28	82.91 ± 10.66	<0.01
**SBP (mmHg)**	139.37 ± 18.90	145.60 ± 19.59	<0.01
**Creatinine (umol/L)**	72.85 ± 13.2	73.25 ± 14.2	0.52

AF, atrial fibrillation; FP , fat percentage; AFP, arm fat percentage; TFP, trunk fat percentage; LFP, leg fat percentage; BMI, body mass index; MET, the metabolic equivalent task; DBP, diastolic blood pressure; SBP, systolic blood pressure; IQR, interquartile range.

Associations between total and regional fat mass and incident AF

The associations between total and regional fat mass and AF risk are presented in **Table 2**. There was a robust and statistically significant association between LFP and FP with a reduced risk of AF across all models. Following multivariable adjustment, including BMI, each standard deviation (SD) increase in LFP was linked to a notable 16% lower risk of AF (hazard ratio [HR]: 0.84, 95% confidence interval [CI]: 0.82, 0.85, P < 0.01, Model 2). In comparison, the association between FP and AF was weaker. Each SD increase in FP was associated with a 10% lower risk of AF (HR: 0.90, 95% CI: 0.89, 0.92, P < 0.01, Model 2). However, AFP and trunk fat percentage TFP exhibited only marginal associations with a lower incidence of AF (AFP, HR: 0.98, 95% CI: 0.96, 1.00; TFP, HR: 0.96, 95% CI: 0.94, 0.97, Model 2).

**Table 2:** Association between total and regional fat percentage and AF incidence.

	Model 1	Model 2
	HR (95%CI)	HR (95%CI)
FP		
**Q1**	Ref	Ref
**Q2**	0.87 (0.84,0.90)	0.86 (0.83,0.89)
**Q3**	0.93 (0.89,0.98)	0.84 (0.81,0.88)
**P for trend**	<0.01	<0.01
**Per SD increase**	0.92 (0.90,0.94)	0.90 (0.89,0.92)
AFP		
**Q1**	Ref	Ref
**Q2**	0.90 (0.87,0.93)	0.89 (0.86,0.92)
**Q3**	0.96 (0.92,1.00)	0.93 (0.89,0.97)
**P for trend**	0.09	0.01
**Per SD increase**	1.00 (0.98,1.03)	0.98 (0.96,1.00)
TFP		
**Q1**	Ref	Ref
**Q2**	0.89 (0.86,0.92)	0.88 (0.86,0.91)
**Q3**	0.93 (0.90,0.97)	0.90 (0.87,0.94)
**P for trend**	<0.01	<0.01
**Per SD increase**	0.97 (0.96,0.99)	0.96 (0.94,0.97)
LFP		
**Q1**	Ref	Ref
**Q2**	0.82 (0.79,0.84)	0.81 (0.78,0.84)
**Q3**	0.75 (0.72,0.78)	0.73 (0.70,0.76)
**P for trend**	<0.01	<0.01
**Per SD increase**	0.84 (0.83,0.86)	0.84 (0.82,0.85)

Model 1: age, race, sex, BMI.

Model 2: adjusted for age, sex, race, myocardial infarction, diabetes, heart failure, diet score, smoking, and alcohol, MET, systolic blood pressure, diastolic blood pressure, medication for hypertension, BMI.

AF, atrial fibrillation; FP, fat percentage; AFP, arm fat percentage; TFP, trunk fat percentage; LFP, leg fat percentage; SD, standard deviation.

**Figure 1:**
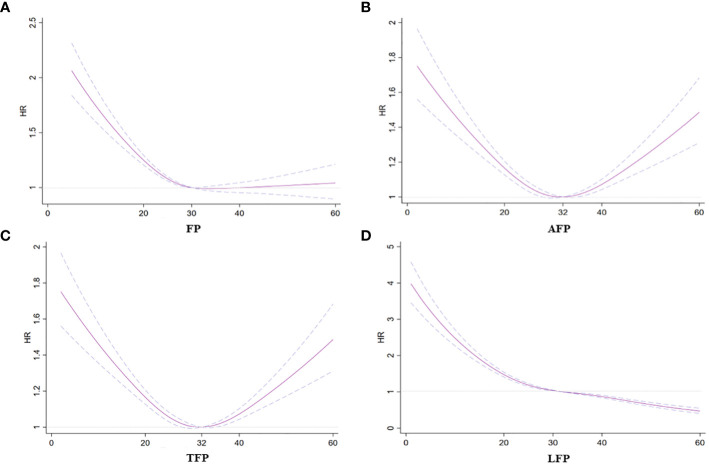
Association between fat mass and regional fat mass percentage with incident AF. **(A)** FP and AF. **(B)** AFP and AF. **(C)** TFP and AF. **(D)** LPF and AF. Hazard ratios are indicated by solid lines and 95% CIs are indicated by a dummy line.

The categorical analysis and restricted cubic splines demonstrated a nonlinear relationship between total and regional fat and incidence of AF after adjusting for confounding factors (**Table 2**; **Figure 1**). Specifically, a U-shaped relationship emerged for AFP and TFP in relation to the incidence of AF (P for nonlinear trend < 0.01; **Figures 1B, C**). The plot illustrated a substantial risk reduction within the lower range of AFP or TFP, reaching the lowest risk of approximately 27% for AFP and 32% for TFP, followed by a slow increase for AFP and a sharp increase for TFP thereafter. Additionally, a negative association was observed between FP and LFP and AF risk. The risk of AF sharply decreased to 30% for FP and 32% for LFP, then decreased slowly for LFP, and did not decrease for FP (P for nonlinear trend < 0.01, **Figures 1A, D**).

### Associations between VAT, ASAT, TTF, and AF

**Figure 2:**
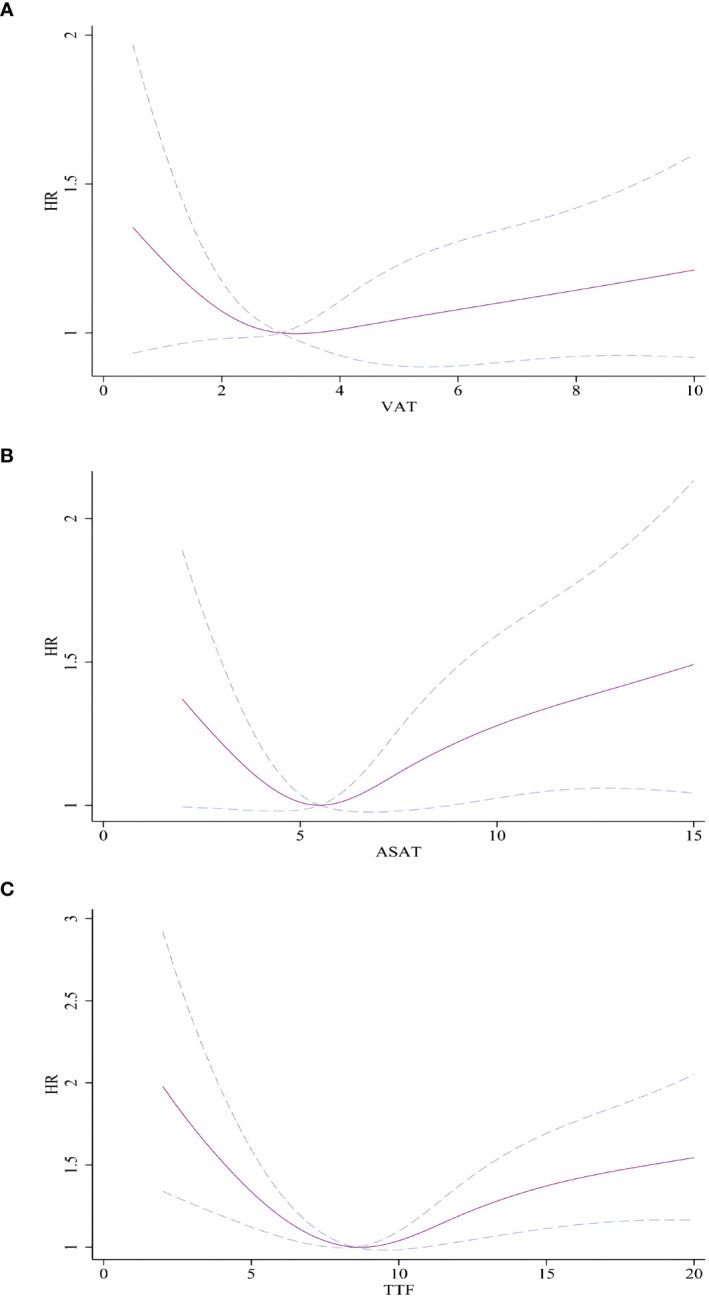
Association between VAT, ASAT, and TTF and with incident AF. **(A)** VAT and AF. **(B)** ASAT and AF. **(C)** TTF and AF. Hazard ratios are indicated by solid lines and 95% CIs are indicated by a dummy line. AF, atrial fibrillation; VAT, Visceral adipose tissue volume; ASAT, Abdominal subcutaneous adipose tissue volume; TTF, total trunk fat volume.

We did not observe statistical associations between VAT, ASAT, and TTF and AF incidence using ASAT, VAT, and TTF as continuous variables (**Supplementary Table 1**). Restricted cubic splines demonstrated a nonlinear relationship between VAT, ASAT, and TTF and incidence of AF after adjusting for confounding factors. A U-shaped relationship was found between VAT, ASAT, and TTF and AF incidence (P for nonlinear trend<0.01, **Figure 2**).

### Sensitivity and subgroup analyses

Our findings remained robust in several sensitivity analyses. The association between regional fat mass and the incidence of atrial fibrillation AF did not change when using AFI, TFI, and LFI instead of AFP, TFP, and LFP (**Supplementary Table 2**). An increase in LFI was associated with a lower risk of AF, and AFP and TFP showed marginal associations with the incidence of AF. Furthermore, when applying the competing risk model instead of the Cox proportional hazard model, the results remained robust (**Supplementary Table 3**). We compared the total and regional fat percentages in participants with AF and matched non-AF participants. Participants with AF had lower LFP and FP percentages (**Supplementary Table 4**). Subgroup and interactive analyses revealed that sex moderated the association between total and regional fat mass and AF incidence (**Supplementary Figure 2**). The protective role of fat mass was weaker in female participants.

## Discussion

In this large, population-based, prospective cohort study including more than 480,000 participants and 29,658 AF outcomes, fat mass, mainly leg fat mass, was associated with a lower risk of AF after adjusting for BMI and traditional risk factors. A U-shaped relationship was observed between arm fat, trunk fat (including VAT and ASAT), and the risk of atrial fibrillation (AF).

Consistent with our findings, Azarbal et al. found that fat mass is associated with a lower risk of AF in postmenopausal women (5). However, Frost et al. showed contrasting results, indicating that higher fat mass was linked to an increased risk of AF (HR: 1.29 per standard deviation increment in fat mass) (4). The controversy in these findings may be attributed to the confirmed correlations between LBM, fat mass, and other anthropometric parameters, as highlighted in our and previous studies (4). Recent investigations have emphasized LBM as the primary anthropometric risk factor for AF (5, 6), suggesting that individuals with higher fat mass may have higher proportions of LBM, contributing to an elevated AF risk. Therefore, the crucial aspect in this context involves the systematic mutual adjustment of relevant anthropometric measures associated with fat mass. Both our study and that by Azarbal et al. included BMI adjustment, assuming that all participants had similar body shapes, although this assumption may be more suited for elucidating methodological mechanisms than offering a clear biological interpretation.

This study represents the first investigation of arm and leg fat mass in relation to atrial fibrillation (AF) risk within a large prospective cohort. The unique findings suggest that the associations between fat mass and AF risk are region-specific, reflecting diverse fat masses in different regions with distinct inflammatory and metabolic functions (14). Our present study expand prior research that has demonstrated associations between fat mass and AF risk factors. Previous study have left gap in our understanding the association between regional fat mass and AF risk in prospective cohort studies, particularly in determining whether additional measurements of regional fat mass can further discriminate participants at high risk of AF in addition to BMI. The varying connections between body fat and cardiovascular risk factors or events across different regions make sense, considering the distinct fat depots in the upper and lower body, each with unique biological functions. Numerous potential mechanisms contribute to these region-specific associations, such as differences in adipose inflammation severity, lipid storage and turnover, release of adipokines, and endocrine effects (14–17). Inflammation and metabolic dysregulation are established mechanisms contributing to the development and progression of AF. Inflammation plays a pivotal role in the initiation and perpetuation of AF by promoting atrial structural remodeling, fibrosis, and electrical instability. Metabolic disturbances, such as insulin resistance and dyslipidemia, have been linked to atrial electrical and structural remodeling, creating a substrate conducive to AF (17, 18). The physiological functions of beige fat extend beyond metabolic regulation, encompassing its role in modulating systemic inflammation and metabolic homeostasis. By virtue of its ability to enhance energy expenditure and improve lipid and glucose metabolism, beige fat activation may mitigate the inflammatory and metabolic perturbations that underlie AF pathogenesis (19). Moreover, recent evidence suggests that beige fat-derived factors, such as adipokines and thermogenic mediators, possess anti-inflammatory and cardioprotective properties (20). Lower limb fat typically contains more beige fat, which is associated with low systemic inflammation and healthy metabolism. These factors may directly influence atrial electrophysiology and remodeling processes, thereby attenuating the susceptibility to AF.

In contrast, a U-shaped relationship was identified between trunk fat mass (including VAT and ASAT) and AF risk in this study. Interestingly, ASAT did not exhibit a protective role, as both high volumes of ASAT and VAT were associated with a higher risk of AF. This aligns with findings from Fox et al., who also observed that elevated levels of both VAT and ASAT were linked to metabolic disorders (20). The present study did not find an exact association While the study did not find a clear association between arm fat mass and AF risk, AFP showed a marginal association with AF incidence, which was not supported by sensitivity analysis. As the arm is not a primary fat storage site and constitutes a small percentage of total fat mass, its impact on inflammation and metabolic factors may be insufficient. However, a U-shaped relationship was noted between arm fat and AF incidence, suggesting that arm fat mass may serve as an indicator of nutritional status (21). Both malnutrition and overnutrition have associations with metabolic disorders, potentially contributing to a higher risk of AF. Further research is warranted to establish the causal association.

### Study strength and limitations

Our study has a multitude of strengths. The incidence of AF is very low in the general population (6.0% in our study). Previous studies with limited samples might have been underpowered to verify the association between total or regional fat distribution and incident AF. Our study included approximately 500,000 participants with a long-term follow-up period (12.1 years), and approximately 30,000 participants developed AF. The sample size was sufficiently large to evaluate the association and conclusively examine sex or race interactions. We used different indicators of regional fat distribution and statistical methods to verify the robustness of our findings. These findings have important clinical and public-health implications. As a simple, effective, and economic method, BIA can further improve risk stratification and reduce the incidence of AF. Furthermore, our findings prompt a reevaluation of obesity management strategies. Traditional measures of obesity, such as BMI, may not capture the nuances of fat distribution that influence AF susceptibility. Clinicians managing obesity patients should consider evaluating regional fat distribution alongside BMI to obtain a more comprehensive assessment of AF risk. Tailoring interventions based on regional fat distribution could lead to more effective strategies for reducing AF incidence among obese individuals.

On the other hand, our study also has several limitations. First, residual confounding is always an issue that cannot be averted in observational studies; however, we believe that residual confounding was a minor issue in the present study, as the relative confounding of AF incidence was fully adjusted. Furthermore, the adjustments had little effect on effect size. Second, the regional fat percentage or index was measured using BIA rather than DXEA/CT/MRI, although relevant studies have demonstrated that BIA has comparable accuracy to that of DEXA (11). Third, epicardial fat is of particular interest in AF due to its proximity to the myocardium and potential implications for cardiovascular health (22). It’s noteworthy that bioimpedance analysis (BIA) cannot directly measure epicardial fat. Fourth, The Biobank study indeed did not measure adipokines secreted by adipose tissue, a factor of considerable relevance in understanding the association between regional fat distribution and AF risk (22).

## Conclusion

In conclusion, fat mass, mainly leg fat mass, was associated with a lower risk of AF after adjusting for BMI and traditional CVD risk factors. ASAT did not exert protective effects.

## Data availability statement

The raw data supporting the conclusions of this article will be made available by the authors, without undue reservation.

## Ethics statement

Ethical approval for the Biobank study was granted by the Northwest Multi-Center Research Ethics Committee(11/NW/0382). The studies were conducted in accordance with the local legislation and institutional requirements. Written informed consent for participation was not required from the participants or the participants’ legal guardians/next of kin in accordance with the national legislation and institutional requirements.

## Author contributions

CW: Writing – original draft, Writing – review & editing. YX: Writing – original draft, Writing – review & editing. ZX: Conceptualization, Writing – review & editing.
