# Supplementary material for: Exploring the association between regional fat distribution and atrial fibrillation risks: a comprehensive cohort study
Source: Front Endocrinol (Lausanne). 2024 Mar 22;15:1367653. doi: 10.3389/fendo.2024.1367653 (PMC10995301; doi:10.3389/fendo.2024.1367653)
Supplement: Supplementary file 1 [file DataSheet_1.docx]

**Supplementary table 1: Association between VAT, ASAT, or TTF measured by MRI and incident AF**

|  | **HR (95%CI)** | |
| --- | --- | --- |
|  | **Model 1** | **Model 2** |
| **VAT** |  |  |
| **Q1** | **Ref** | **Ref** |
| **Q2** | 1.09(0.92,1.29) | 1.02(0.86,1.21) |
| **Q3** | 1.50(1.28,1.75) | 1.31(1.12,1.54) |
| **P for trend** | 0.05 | <0.01 |
| **Per SD increase** | 1.08(1.00,1.17) | 1.05(0.96,1.14) |
| **ASAT** |  |  |
| **Q1** | **Ref** | **Ref** |
| **Q2** | 0.92(0.77,1.09) | 0.90(0.76,1.06) |
| **Q3** | 1.11(0.91,1.36) | 1.08(0.88,1.32) |
| **P for trend** | 0.33 | 0.49 |
| **Per SD increase** | 1.08(0.98,1.19) | 1.07(0.97,1.18) |
| **TTF** |  |  |
| **Q1** | **Ref** | **Ref** |
| **Q2** | 0.88(0.74,1.05) | 0.86(0.72,1.02) |
| **Q3** | 1.22(0.99,1.50) | 1.14(0.93,1.40) |
| **P for trend** | 0.06 | 0.20 |
| **Per SD increase** | 1.11(1.00,1.23) | 1.08(0.97,1.19) |

Model 1: age, race, sex, BMI

Model 2: adjusted for age, sex, race, myocardial infarction, diabetes, heart failure, diet score, smoking, and alcohol, MET, systolic blood pressure, diastolic blood pressure, medication for hypertension, BMI.

AF=atrial fibrillation, VAT=Visceral adipose tissue volume; ASAT=Abdominal subcutaneous adipose tissue volume; TTF=total trunk fat volume;SD=standard deviation.

**Supplementary Table 2: Association between reginal fat index and incident AF**

|  | HR (95%CI) | |
| --- | --- | --- |
|  | **Model 1** | **Model** 2 |
| **FMI** |  |  |
| **Q1** | **Ref** | **Ref** |
| **Q2** | 0.91(0.88,0.94) | 0.87(0.84,0.90) |
| **Q3** | 0.93(0.89,0.98) | 0.88(0.84,0.92) |
| **P for trend** | 0.96(0.94,0.98) | 0.93(0.91,0.95) |
| **Per SD increase** | 0.89(0.86,0.93) | 0.91(0.87,0.95) |
| **AFI** |  |  |
| **Q1** | **Ref** | **Ref** |
| **Q2** | 0.96(0.93,1.00) | 0.93(0.90,0.96) |
| **Q3** | 1.02(0.97,1.07) | 0.97(0.93,1.02) |
| **P for trend** | 1.01(0.98,1.03) | 0.98(0.96,1.00) |
| **Per SD increase** | 1.05(1.02,1.08) | 1.04(1.01,1.07) |
| **TFI** |  |  |
| **Q1** | **Ref** | **Ref** |
| **Q2** | 0.89(0.86,0.92) | 0.87(0.84,0.90) |
| **Q3** | 0.95(0.91,0.99) | 0.91(0.87,0.95) |
| **P for trend** | 0.98(0.96,1.01) | 0.96(0.93,1.01) |
| **Per SD increase** | 0.99(0.96,1.02) | 0.98(0.94,1.01) |
| **LFI** |  |  |
| **Q1** | **Ref** | **Ref** |
| **Q2** | 0.92(0.89,0.95) | 0.86(0.84,0.90) |
| **Q3** | 0.81(0.77,0.86) | 0.78(0.73,0.82) |
| **P for trend** | 0.91(0.88,0.93) | 0.88(0.85,0.90) |
| **Per SD increase** | 0.78(0.75,0.82) | 0.83(0.79,0.86) |

Model 1: age, race, sex, BMI

Model 2: adjusted for age, sex, race, myocardial infarction, diabetes, heart failure, diet score, smoking, and alcohol, MET, systolic blood pressure, diastolic blood pressure, medication for hypertension, BMI.

AF=atrial fibrillation,AFI=arm fat index,TFI= trunk fat index,LFI = leg fat index;SD=standard deviation.

**Supplementary Table 3: Association between total and reginal fat percentage and incident AF using competing regress models**

|  | **Model 1** | | **Model2** | |
| --- | --- | --- | --- | --- |
|  | HR (95%CI) | P value | HR (95%CI) | P value |
| **FP per SD** | 0.92(0.90,0.94) | <0.01 | 0.89(0.88,0.90) | <0.01 |
| **AFP per SD** | 1.00(0.98,1.03) | 0.31 | 0.98(0.95-1.01) | 0.38 |
| **TFP per SD** | 0.97(0.95,1.02) | 0.06 | 0.97(0.93-1.01) | 0.27 |
| **LFP per SD** | 0.84(0.83,0.86) | <0.01 | 0.85(0.80-0.90) | <0.01 |

Model 1: age, race, sex, BMI

Model 2: adjusted for age, sex, race, myocardial infarction, diabetes, heart failure, diet score, smoking, and alcohol, MET, systolic blood pressure, diastolic blood pressure, medication for hypertension, BMI.

AF=atrial fibrillation,FP =fat percentage, AFP= arm fat percentage, TFP=trunk fat percentage, LFP=leg fat percentage, SD=standard deviation.

**Supplementary Table 4: Association between total and reginal fat percentage and incident AF after the propensity score matching**

|  | AF | Non-AF | P value |
| --- | --- | --- | --- |
| **FP** | 30.7±8.5 | 31.6±8.7 | <0.01 |
| **AFP** | 29.9±10.5 | 29.9±10.3 | 0.25 |
| **TFP** | 32.3±8.1 | 32.2±7.7 | 0.10 |
| **LFP** | 30.7±10.8 | 31.1±10.7 | <0.01 |

AF=atrial fibrillation,FP =fat percentage, AFP= arm fat percentage, TFP=trunk fat percentage, LFP=leg fat percentage, SD=standard deviation.

**Supplemental Figure 1: Correlation matrix of body composition characteristics. P-value for all comparisons <0.01.**


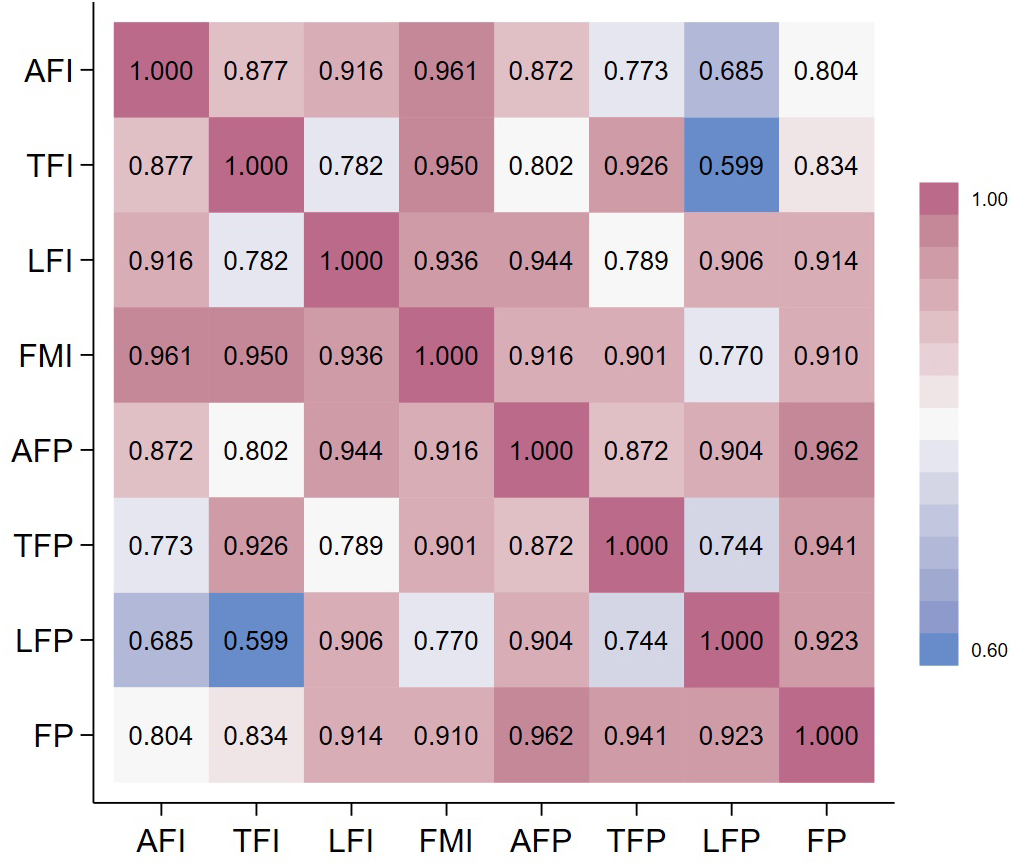


AFI=arm fat index,TFI= trunk fat index,LFI = leg fat index,AFP= arm fat percentage, TFP=trunk fat percentage, LFP=leg fat percentage, FP =fat percentage.

**Supplementary Figure 2: Subgroup analysis**


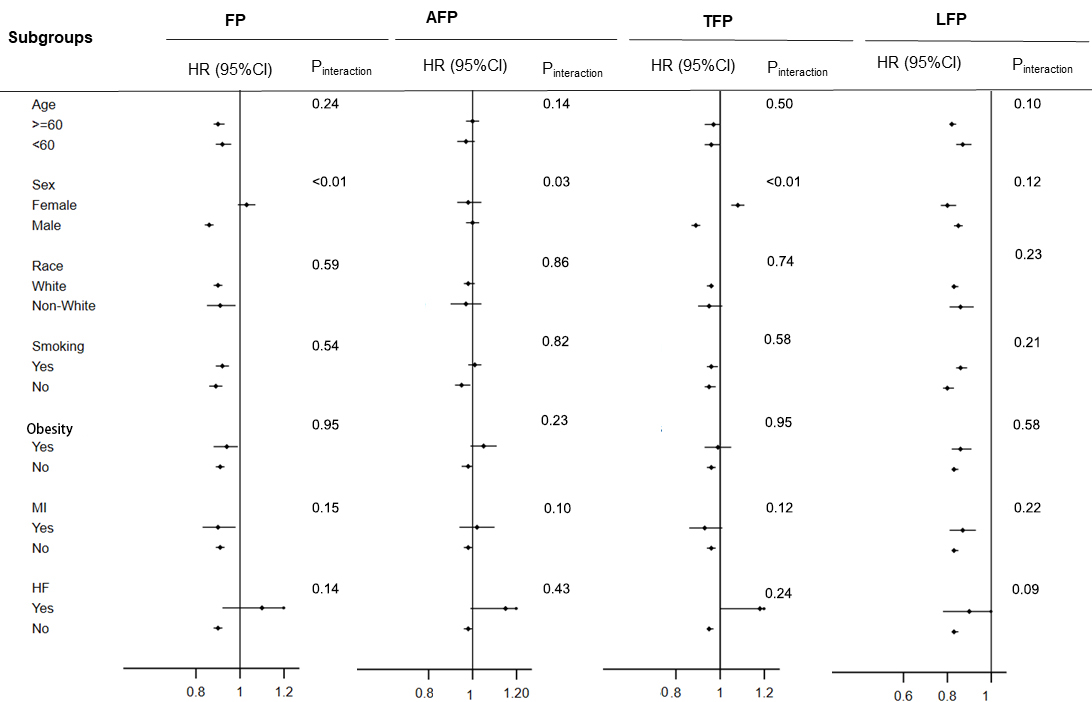


Hazard ratios per one SD increase in fat percentage and regional fat mass percentage for AF. Each stratification was adjusted for all factors in Model 2. FP =fat percentage, AFP= arm fat percentage, TFP=trunk fat percentage, LFP=leg fat percentage, SD=standard deviation.
